# Designing and identifying β-hairpin peptide macrocycles with antibiotic potential

**DOI:** 10.1126/sciadv.ade0008

**Published:** 2023-01-11

**Authors:** Justin R. Randall, Cory D. DuPai, T. Jeffrey Cole, Gillian Davidson, Kyra E. Groover, Sabrina L. Slater, Despoina A. I. Mavridou, Claus O. Wilke, Bryan W. Davies

**Affiliations:** ^1^ Department of Molecular Biosciences, University of Texas at Austin, Austin, TX, USA.; ^2^ Department of Integrative Biology, University of Texas at Austin, Austin, TX, USA.

## Abstract

Peptide macrocycles are a rapidly emerging class of therapeutic, yet the design of their structure and activity remains challenging. This is especially true for those with β-hairpin structure due to weak folding properties and a propensity for aggregation. Here, we use proteomic analysis and common antimicrobial features to design a large peptide library with macrocyclic β-hairpin structure. Using an activity-driven high-throughput screen, we identify dozens of peptides killing bacteria through selective membrane disruption and analyze their biochemical features via machine learning. Active peptides contain a unique constrained structure and are highly enriched for cationic charge with arginine in their turn region. Our results provide a synthetic strategy for structured macrocyclic peptide design and discovery while also elucidating characteristics important for β-hairpin antimicrobial peptide activity.

## Teaser

Synthetic identification of membrane selective beta-hairpin antimicrobial peptides enriched for arginine in their loop region.

## INTRODUCTION

The stability and broad functionality of macrocyclic peptides makes them a promising area for drug development; however, there are few well-characterized strategies for their identification. Current de novo design remains challenging, especially for those with β-hairpin structure (*1*–*4*). They often lack sufficient interstrand interactions to form stable conformations and their β strands promote association, which can lead to aggregation in solution (*2*–*4*).

Antibiotics provide an excellent example of macrocyclic peptide drug value, and new discovery strategies are urgently needed (*5*). Macrocyclic β-hairpin antimicrobial peptides (β-AMPs) have recently gained popularity, with two such antibiotics having proceeded into clinical evaluation (*6*–*8*). These β-AMPs act primarily through bacterial membrane permeabilization, allowing them to overcome most mechanisms of bacterial drug resistance and also access and inhibit essential processes within the gram-negative cell envelope (*9*, *10*); however, known examples are exceedingly rare. This makes it difficult to understand how β-AMP sequence determines structure and function (*11*–*13*). Established strategies for de novo β-hairpin peptide design limit the use of amino acids important for antibacterial activity and include residues that increase mammalian cell toxicity, which is detrimental for therapeutic development (*1*, *11*).

Here, we describe the design, screening, and analysis of a synthetic macrocyclic β-hairpin peptide library using new strategies and technologies. Our results expand our understanding of β-AMP sequence-activity relationships and provide a route for their design and discovery.

## RESULTS

### Design of a synthetic macrocyclic β-hairpin peptide library

**Fig. 1.**
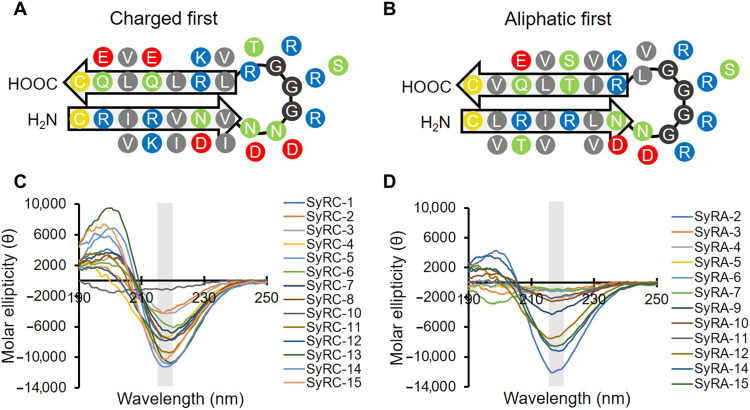
SynCH peptides show β-hairpin secondary structure. Diagrams showing the designed structure and residue position of the charged first (**A**) and aliphatic first (**B**) SynCH libraries. Residues are color-coded by side chain (yellow, cysteine; green, polar; gray, aliphatic; blue, positive; red, negative). (**C** and **D**) Circular dichroism spectra of randomly selected charged first (SyRC) and aliphatic first (SyRA) SynCH peptides. A single minimum between 215 and 220 nm (gray box) is characteristic of β-hairpin secondary structure. Each spectrum is the mean of three technical replicates with background subtracted.

Our design scheme leveraged our recently completed systematic analysis of more than 49,000 β-hairpin motifs in the Protein Data Bank. This analysis identified position-specific amino acid preferences in the strand and turn regions (*14*). Using this information, we designed two ribosomally encoded 20–amino acid cyclic β-hairpin peptide libraries (Fig. 1, A and B, and fig. S1).

These two libraries were intentionally designed to facilitate the development of stable antiparallel β strands with amphipathic faces generated via the periodic alternation of aliphatic and charged/polar residues. We identified and applied a preference for glycine, asparagine, and aspartic acid in or near the turn regions (*14*–*16*) and excluded proline in contrast to canonical β-turn design (*17*–*20*). We also excluded aromatic residues because they promote mammalian toxicity despite their positive effects on β-hairpin structure (*21*). Cysteine was encoded at the N and C termini to potentiate cyclization via a disulfide bridge and prevent fraying. Last, we allowed select polar positions and the loop region to encode for positive residues to promote solubility and mimic AMP characteristics. Using codon variation, we created and combined two synthetic, macrocyclic β-hairpin (SynCH) libraries based on our design scheme, one beginning with a charged residue and the other with an aliphatic residue. This allows different amphipathic characters to occupy different faces relative to the loop region. In total, this pooled library encompassed 196,608 unique peptide sequences with a variety of physiochemical properties (Fig. 1, A and B, and fig. S1).

### SynCH peptides spontaneously fold into macrocyclic β-hairpins

We examined the structure of 30 SynCH peptides at random from the charged first (SyRC) and aliphatic first (SyRA) libraries spanning a range of charge (−0.12 to 4.87) and grand average of hydropathicity (GRAVY) score (−0.72 to 0.41) (table S1). One SyRC peptide and three SyRA peptides could not be synthesized. We used circular dichroism (CD) spectroscopy to determine the secondary structure of each synthesized peptide (Fig. 1, C and D, and table S1). Remarkably, 84.6% of all peptides had a CD spectrum with a single ellipticity minimum between 215 and 220 nm, indicative of antiparallel β-sheet secondary structure (*22*). This is highly uncommon for peptides in aqueous solution. Most require a target interaction to form a β hairpin or other secondary structure (*23*, *24*).

Next, we performed high-resolution liquid chromatography–mass spectrometry (LC-MS) on each peptide to determine whether an intramolecular disulfide bond was present (table S1 and fig. S2). All but three of the randomly selected SynCH peptides examined had a disulfide bond present in the majority of their molecular population. While sequences from the aliphatic first library were less likely to show β-sheet secondary structure (Fig. 1, C and D), they were slightly more likely to have a majority of their molecular population cyclized (table S1). These data together suggest that ~72% of our SynCH peptide library forms a stable β-hairpin secondary structure and are cyclized through an intramolecular disulfide bond in solution.

### Identification of SynCH peptides with antibiotic potential using SLAY

**Fig. 2.**
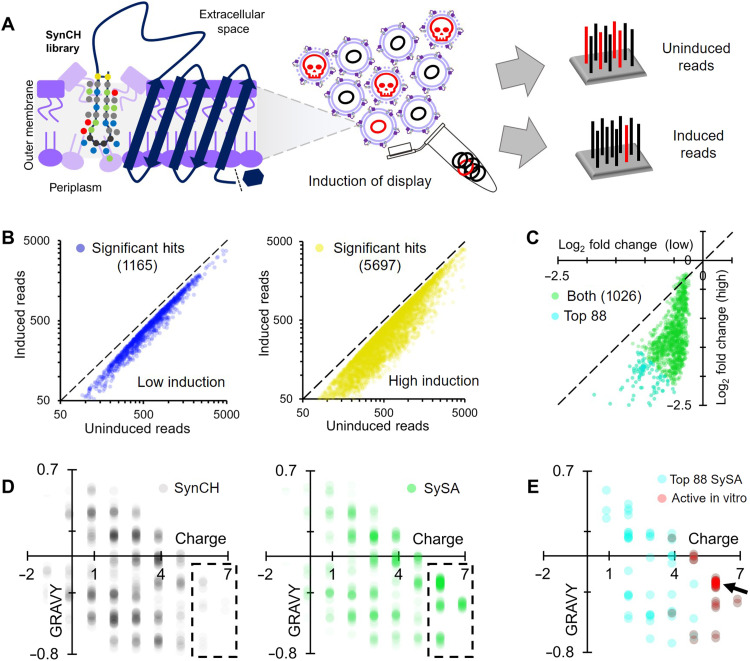
SLAY identifies SynCH peptides with antibacterial potential. (**A**) Workflow of a surface-localized antimicrobial display (SLAY) screen. (**B**) Density scatter plot for SynCH peptides with a significant, negative log_2_ fold change (*P* < 0.05) from SLAY induced at low (left) and high (right) concentrations. (**C**) Density scatter plot of each SynCH peptide with a significant, negative log_2_ fold change in reads (*P* < 0.05) under both induction conditions (SySA). (**D**) Density scatter plots for a randomized subset of the SynCH library (left) and SySA (right). (**E**) Same plot but with the top 88 SySA peptides based on average log_2_ fold change. Those which were verified to have in vitro activity are in red; 18 of 36 are from a single grouping (arrow).

Our randomly selected SynCH peptides were not inherently antibacterial (table S1), so we decided to use a high-throughput genetic platform developed in our laboratory called surface-localized antimicrobial display (SLAY) (*25*, *26*) to screen for antibacterial activity. SLAY functions through the inducible display of a plasmid-encoded peptide library on the gram-negative bacterial cell surface. Next-generation sequencing is then used to generate a log_2_ fold change in peptide sequence read counts between induced and uninduced bacterial cultures (Fig. 2A).

We performed SLAY on our SynCH library at low [15 μM isopropyl-β-d-thiogalactopyranoside (IPTG)] and high (100 μM IPTG) induction concentrations to mimic treating bacteria with two different concentrations of peptide. The low induction (15 μM IPTG) condition identified 1165 such peptides, referred to as “hits” (Fig. 2B, left). The high induction (100 μM IPTG) condition identified substantially more hits (5697) (Fig. 2B, right). Next, we plotted the log_2_ fold change of the hits from both induction concentrations against one another (Fig. 2C). A total of 1026 peptides, or 2.49% of the total number of peptides screened (~41,000), were identified as a hit under both conditions. This group of 1026 peptides are referred to as the SynCH SLAY active group, or “SySA” going forward (Fig. 2C, green). The most promising 88 hits are highlighted (Fig. 2C, cyan).

To see whether there was enrichment of SynCH peptides with a certain charge or hydrophobicity in the SySA group, we plotted the distribution of charge versus GRAVY score for an equal number of randomly selected SynCH peptides and compared them to the SySA group (Fig. 2D). SySA distribution was highly enriched at charges greater than 5.5 relative to the SynCH library, suggesting that cationic charge is important for their antibacterial potential. The top 88 SySA peptides were further enriched toward cationic charge (Fig. 2E, cyan). Eighteen of 36 SySA peptides with verified in vitro activity (detailed below) were within a single distribution grouping (charge of ~5.87 and GRAVY of −0.2 to −0.3) (Fig. 2E, red and arrow). For a complete list of SLAY and SySA information, see data files S1 and S2.

### SySA peptides selectively disrupt bacterial membranes

**Fig. 3.**
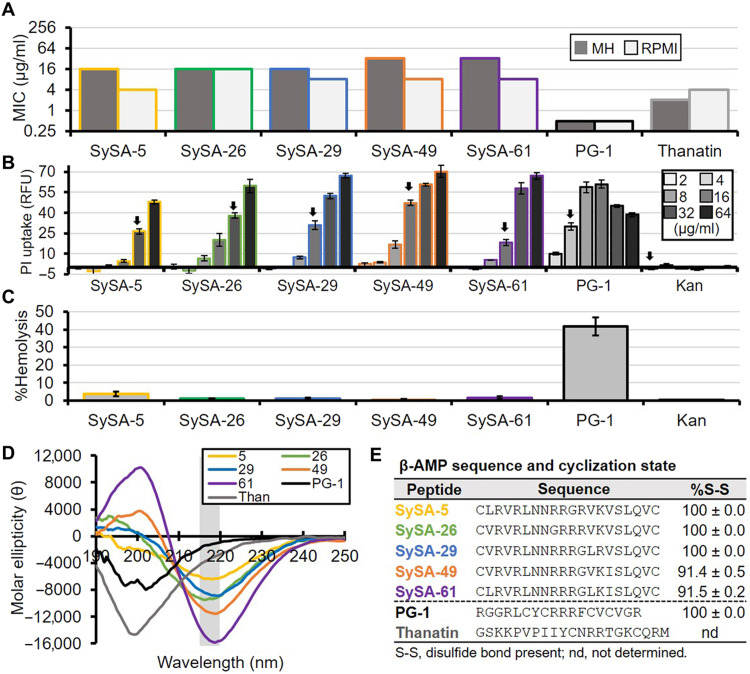
SySA peptide antibiotics demonstrate membrane specificity and have cyclic β-hairpin structure. (**A**) Minimum inhibitory concentration (MIC) of select SySA peptides, Protegrin-1 (PG-1) and Thanatin, against *E. coli* W3110 in Mueller-Hinton (MH) and RPMI 1640 (RPMI) media. Each bar represents the median MIC (*n* = 3). (**B**) Propidium iodide (PI) uptake of *E. coli* W3110 cells treated with serially diluted peptide and kanamycin (Kan). Bars represent the mean; error bars are 1 SD (*n* = 3). Arrows point to the minimum bactericidal concentration (MBC) under these experimental conditions. (**C**) Percent hemolysis relative to treatment with 1% Triton X-100. Bars represent the median, and error bars are 1 SD (*n* = 3). (**D**) CD spectra of peptides. A single minimum between 215 and 220 nm (gray box) is characteristic of β-hairpin structure. Each spectrum is the mean of three technical replicates with background subtracted. (**E**) Table of each peptide sequence and the percentage of the molecular population with an intramolecular disulfide bond. Error bar is 1 SD of technical replicates (*n* = 3).

We chose to chemically synthesize the top 88 SySA peptides as determined by lowest average log_2_ fold change for further biochemical characterization (Fig. 2C, cyan) along with two natural β-AMPs lacking their C-terminal amidation for comparison (Protegrin-1 and Thanatin) (data file S1 and Supplementary Text). We began by determining the minimum inhibitory concentration (MIC) for each SySA peptide against our screening strain, *Escherichia coli* W3110, in standard Mueller-Hinton (MH) broth and the tissue culture media RPMI 1640 (RPMI). MH broth is a standard for antibacterial testing, while RPMI medium better represents salt and buffer conditions found in the body. 44.4% (36 of 81) of the SySA peptides examined were active in vitro in both media, with MICs ranging from 4 to 256 μg/ml (table S2). The vast majority of active peptides were from the aliphatic first library. All active SySA peptides had a charge greater than 3.8, with 78% having a charge greater than 5.8. Interestingly, 18 of the 36 peptides had a charge of 5.87 and a GRAVY score between −0.2 and −0.3 (Fig. 2E, arrow, and table S2, bold). This grouping contained many of the most potent SySA peptides, so we chose five (MICs of 4 to 32 μg/ml) to investigate further (Fig. 3A). Overall, this set of peptides was less potent than Protegrin-1 and Thanatin, whose MICs ranged from 0.5 to 4 μg/ml (table S2). This is not surprising considering that our SySA peptides have not been optimized for antibacterial activity, while natural β-AMPs have evolved their activity over millennia. For all peptide MICs, see data file S1.

We were curious whether SySA peptides functioned via membrane disruption, like Protegrin-1, or an intracellular mode of action. To investigate, we measured the amount of propidium iodide (PI) uptake caused by our select SySA peptides and compared them to Protegrin-1 and two intracellular acting antibiotics, kanamycin and ciprofloxacin (Fig. 3B and fig. S3). PI fluorescence occurs upon DNA binding, indicating that both the outer and inner membrane have been permeabilized (fig. S3). We treated *E. coli* with twofold serial dilutions of antimicrobial and observed PI uptake by relative fluorescence units (RFUs). All five of our SySA peptides showed high levels of PI uptake similar to Protegrin-1 near the minimum bactericidal concentration (MBC) of each determined under these conditions (Fig. 3B, arrows). SySA peptides also caused release of green fluorescent protein (GFP) expressed in the cytoplasm of *E. coli* cells into the media upon peptide treatment at concentrations below each’s MBC determined at the same cell density (fig. S4). The MIC of each peptide was additionally examined in cation-adjusted MH (MHII). The addition of divalent cations (i.e., Ca^2+^ and Mg^2+^) increases cell surface integrity through lipopolysaccharide (LPS) crosslinking (*27*). All SySA peptides examined showed an 8- to 16-fold increase in MHII MIC relative to standard MH, where only a twofold increase was observed with kanamycin and ciprofloxacin (data file S1). Together, the above data strongly support outer and inner membrane permeabilization as the primary mode of action for SySA peptides.

Most natural β-AMPs like Protegrin-1 are also highly toxic to mammalian cells because they lack membrane specificity. For this reason, we examined each SySA peptide for its toxicity using a hemolysis assay measured as a percentage of red blood cells lysed by peptide (300 μg/ml) compared to full lysis with a detergent (table S2). Our select SySA peptides showed negligible hemolysis (<3.73%) of red blood cells, like SySA-49, showing virtually no hemolysis (0.29 ± 0.49%) (Fig. 3C and table S2). For comparison, Protegrin-1 lysed 41.8 ± 5.03% of red blood cells and kanamycin, which does not function via membrane disruption, lysed only 0.1 ± 0.03% (Fig. 3C). These results suggest that active SySA peptides function through membrane disruption but demonstrate bacterial membrane selectively. This is an uncommon characteristic for members of the β-AMP class. For a full list of peptide hemolysis, see data file S1.

Last, we questioned whether the naïve peptide sequences discovered through our screen could be optimized to improve their potency, so we generated a 27-peptide optimization library around our most potent peptide (SySA-5) using previously described design principles (*24*). Most variants showed fourfold or greater potency against a multidrug-resistant strain of *Acinetobacter baumannii* with little or no increased toxicity (table S3). One variant, SySA-5.17, was as potent as Protegrin-1 in 100% fetal bovine serum with over 10-fold less toxicity. SySA-5.17 also showed broad-spectrum activity against both gram-positive and gram-negative pathogens, including colistin-resistant strains, suggesting that its activity is not outer membrane or LPS specific (tables S4 and S5). For a full description of optimization variants, see the Supplementary Text.

### SySA peptides have a constrained cyclic β-hairpin structure

Natural antibacterial peptides, including β-AMPs, generally require membrane or membrane mimics like LPS to form α-helical or β-hairpin secondary structures (*23*, *24*). For example, our natural β-hairpin peptide controls have a single molar ellipticity minimum at 200 nm in phosphate buffer alone, consistent with a random coil secondary structure (Fig. 3D) (*22*); however, the CD spectra of all but one of the 36 active SySA peptides (SySA-45) have a single minimum between 215 and 220 nm consistent with a β-hairpin secondary structure (Fig. 3D, table S2, and fig. S5A). This suggests that SySA peptides are more conformationally constrained than many natural β-AMPs. However, this alone does not dictate antibacterial activity because most of our inactive SySA peptides are also conformationally constrained (fig. S5B). Conformational changes in β-AMP structure upon membrane interaction have been implicated in causing mammalian cytotoxicity (*28*). This could help explain the low hemolysis observed with our SynCH peptides. The molar ellipticity of the SySA-49 CD spectrum was equivalent at increased and reduced concentrations (fig. S5C), suggesting that peptide aggregation is not occurring at these concentrations in solution.

Next, we looked to confirm that our most active SySA peptides were also cyclized via a disulfide bond by using LC-MS analysis. All five of the most potent SySA peptides also had a majority of their molecular population participating in a disulfide bond (Fig. 3E). Three of these peptides (SySA-5, SySA-26, and SySA-29) were 100% cyclized in solution. These data, combined with our CD analysis, strongly suggest that active SySA peptides have a macrocyclic β-hairpin structure, which is conformationally constricted, a unique feature for members of the β-AMP class.

### Machine learning identifies features important for antibacterial activity

**Fig. 4.**
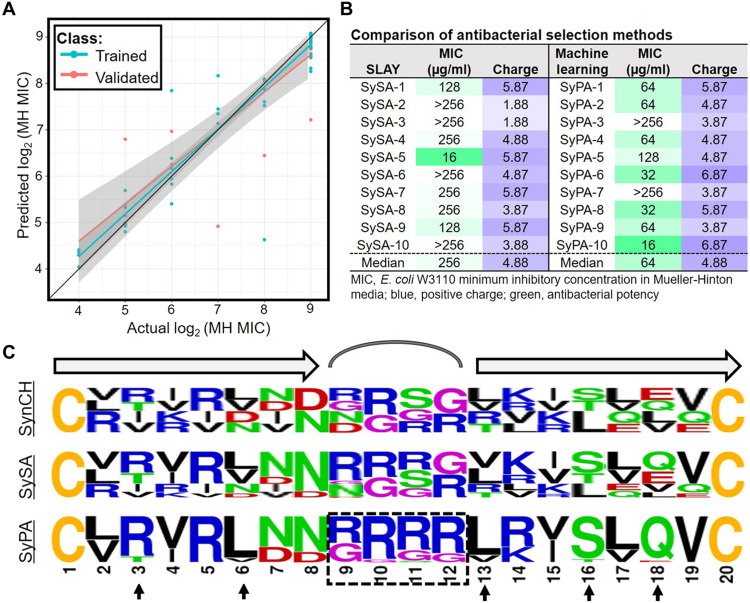
Machine learning identifies features important for activity. (**A**) Actual versus predicted log_2_ MIC models produced via training and validation of the machine learning algorithm. (**B**) Table listing the MIC and charge of peptides identified with SLAY (left panel) or the machine learning (right panel). (**C**) Residue frequencies at each position of 100 SynCH peptides (top), the top 100 SySA peptides (middle), and the top 100 SyPA peptides (bottom). Residues are color-coded based on side-chain properties: blue, basic; red, acidic; green, polar; black, aliphatic; purple, no side chain; yellow, sulfur containing.

The design of the SynCH library reliably produced cyclized β-hairpins with low/no hemolytic activity, but identifying sequence features responsible for antibacterial activity was more challenging. To overcome this hurdle, we trained a machine learning algorithm to predict peptide potency with 80% of our SySA biochemical data and used the remaining 20% for validation. For a detailed description of our model, please see the Supplementary Text. The final trained model was able to explain 90% of the variation (*R*^2^ = 0.90, *P* < 2.2 × 10^−16^) in the training dataset and 78% of the variation (*R*^2^ = 0.78, *P* = 2.5 × 10^−8^) in the validation dataset. This implies that the trained model accurately predicted the activities of SySA sequences set aside for validation (Fig. 4A). A predictive potency score was then generated using this algorithm for all 196,608 peptide sequences in the SynCH library (data file S2).

To assess the accuracy of our predictive modeling, we randomly selected 20,000 SynCH peptides excluded from our SLAY analysis and chose the 10 with the lowest predicted potency score (SynCH predicted active, or “SyPA”). We had these 10 peptides commercially synthesized and measured their MIC in MH against *E. coli* W3110. Remarkably, 8 of 10 SyPA peptides were active, with MIC values ranging from 16 to 128 μg/ml (median MIC, 64 μg/ml; Fig. 4B). This was a vast improvement to our top 10 SLAY screening results, where only 6 of 10 peptides were active and less potent overall (median MIC, 256 μg/ml; Fig. 4B). One predicted peptide (SyPA-10) was as potent in MH media as any of the top 81 SySA peptides that we examined. Furthermore, our modeling also identified active peptides with charges less than 5.8 (SyPA-2, SyPA-4, SyPA-5, and SyPA-9), and these peptides were more potent than peptides of similar charge identified using SLAY. For a full list of SyPA data, see data file S1.

To explore the sequence features most important for predicting antibacterial potency, we plotted the residue frequency at each position for 100 random SynCH peptides and compared them to the top 100 peptides selected by SLAY (SySA) and predicted by machine learning (SyPA) (Fig. 4C) The SySA peptides had few obvious differences from SynCH other than a slight preference for the aliphatic first library. However, several sequence features were clearly enriched in the SyPA group (Fig. 4C, bottom). SyPA peptides were almost exclusively from the aliphatic first library possibly because of a greater potential for arginine in their loop region, which was also clearly enriched. Arginine was also highly enriched at position three. In addition, leucine was highly preferred over valine at positions 6 and 13. Last, serine and glutamine were vastly favored over threonine and glutamate at positions 16 and 18, respectively.

## DISCUSSION

The goal of peptide design is to develop de novo sequences with an intended structure and activity. Here, we successfully designed and produced a large peptide library with predominantly macrocyclic β-hairpin structure and identified those with antibiotic activity. Our design lacked several features commonly used in β-hairpin peptide design including the use of proline in the loop region and aromatic residues like tryptophan in the β-sheet regions (*1*). In contrast, arginine was highly tolerated in both the loop region and β sheets. We found this remarkable, as the loop sequence is considered critical for final β-hairpin stability (*29*), yet arginine is not often considered in loop design.

The β-AMPs identified from our library killed through membrane disruption but were not hemolytic. This membrane selectivity is highly unusual for β-AMPs (*12*) and warrants further study. It is possible that the constrained structure of SynCH peptides helps contribute to this selectivity. Conformational change has been shown to increase cytotoxicity in variants of Protegrin-1 (*28*). However, the lack of aromatic residues in SynCH peptides could also contribute. SySA-5.17 was found to have broad-spectrum activity against both gram-negative and gram-positive bacterial pathogens and colistin-resistant mutants (tables S4 and S5). This suggests that its activity is not dependent on the presence of an outer membrane or impacted by lipid A phosphate modification. We hypothesize that SySA membrane specificity may result from binding of anionic phospholipids found primarily in bacterial membranes such as phosphatidylglycerol and/or cardiolipin. This hypothesis will be pursued in future studies.

Our machine learning algorithm accurately predicted active SynCH peptides with greater accuracy than our cell-based approach and helped identify several sequence features associated with antibacterial potency. Most prominent was the enrichment of arginine, especially within the loop region, which could also explain a preference for the aliphatic first library. This enrichment increases the overall charge of the peptide and is likely important for interaction with the negatively charged outer membrane. Reasons for the enrichment of residues at other positions are less clear, but these preferences could be related to preferred interstrand contacts between antiparallel β sheets.

We are excited about the future possibilities of pairing functional cell-based peptide screening technology with machine learning strategies, especially for antibiotic discovery. We believe that as more antibacterial peptide data become available through synthetic screening technology, machine learning may be able to predict antibacterial activity de novo, bypassing the need for human design and functional screening entirely.

## MATERIALS AND METHODS

### SynCH library cloning

Detailed methods for library creation have been previously reported (*25*, *26*). Briefly, the two library inserts were generated by polymerase chain reaction (PCR) using forward primer oJR557 with reverse primers oJR560 and oJR561 encoding each library. The 2x(NR)tether gBlock was used as the template. Both inserts and the pMMBEH67_lpp_ompA vector were digested with Kpn I and Sal I independently, and the two libraries were ligated overnight at 4°C using T4 ligase. The two ligated libraries were cleaned and transformed into *E. coli* 10-β competent cells (New England Biolabs) and plated on large bioassay dishes (250 mm) filled with LB agar supplemented with carbenicillin (75 μg/ml) and grown overnight. The next morning, colonies were counted, scraped, pooled, aliquoted, and frozen in 10% glycerol for long-term storage. One milliliter of pooled cells was thawed and maxi-prepped to obtain the combined SynCH plasmid library. This library was then transformed into *E. coli* W3110 competent cells for further analysis via SLAY (see below).

### Peptide synthesis

All peptides used in this work were synthesized commercially by GenScript’s custom peptide synthesis service. Each peptide goes through reversed-phase high-performance LC and MS quality control analysis to confirm purity and molecular weight. Lyophilized peptides were resuspended in water at 10 μg/ml. Equivalent peptides synthesized from different batches showed similar biochemical qualities. A full list of all of the peptides used and their purity can be found in data file S1.

### CD spectroscopy

Stock peptides were diluted in 10 mM potassium phosphate (pH 7.4) to 200 μg/ml in a volume of 200 μl. Samples were incubated 1 to 2 hours at room temperature and then analyzed using a Jasco-815 CD spectrometer with a 0.1-cm path-length quartz cuvette at the Targeted Therapeutic Drug Discovery & Development Program Core at the University of Texas at Austin. The CD spectra were collected using far ultraviolet spectra (190 to 250 nm) with background corrected for phosphate-buffered saline (PBS) alone. Ellipticity was converted from mdeg to molar ellipticity. Reported spectra are an average of three separate spectra obtained from the same sample and adjusted for molar concentration.

### High-resolution MS

Stock peptides were diluted into PBS (pH 7.4) at 0.1 mg/ml in a volume of 150 μl and placed into 2-ml autosampler vials with small volume inserts (Fisherbrand, 03-391-8). Samples were separated by a C8 LC column, and an extracted ion chromatogram was generated for the most prevalent charge state isotopes. Mass spectra were generated for each extracted LC peak using an Agilent Technologies 6546 Accurate Mass quadrupole orthogonal acceleration–time-of-flight LC-MS instrument. Analysis was performed using Agilent MassHunter Qualitative software v10. The isotope distribution for each LC peak from each sample was compared to predicted distributions created using Agilent’s Isotope Distribution Calculator. Percentages of molecules with disulfide bonds was determined by comparing the area under the curve for each LC peak containing a disulfide bond over the total area of all relevant peaks (sum area %) and reported as a mean of three technical replicates ± 1 SD.

### Minimum inhibitory concentration

MICs were performed by a modifiedClinical and Laboratory Standards Institute) procedure. Stock peptides were diluted to 2.56 mg/ml in 50 μl of 0.01% acetic acid containing 0.2% bovine serum albumin. Peptide was then serially diluted twofold in this solution, and 10 μl of each dilution was added to a polypropylene 96-well plate (Corning, ref. no. 3879) in triplicate. Separately, *E. coli* W3110 or *A. baumannii* AB5075 was grown overnight in 5 ml of standard MH at 37°C. Cells from these cultures were diluted to a concentration of 5 × 10^5^ to 6 × 10^5^ cells/ml in either standard MH, MHII, RPMI, or fetal bovine serum, and 90 μl was added to each well of the 96-well plate containing diluted peptide. Plates were wrapped twice in parafilm and incubated at 37°C for 18 to 24 hours. Wells were examined by eye against wells with no bacteria for signs of growth. MICs were reported as the minimum concentration with no observable growth. In cases where triplicate samples differed, the concentration supported by the median of the three replicates was reported.

### Minimum bactericidal concentration

MBC was carried by the same MIC procedure performed in either PBS supplemented with 50 mM glucose at a final OD_600_ (optical density at 600 nm) of 0.05 (PI uptake) or MH at a final OD_600_ of 0.8 (GFP release). After 18 to 24 hours of growth at 37°C, 5 μl of each well was spotted on LB agar and placed at 37°C overnight. The lowest concentration of peptide resulting in no cell growth was listed as the MBC. In cases where triplicate samples differed, the concentration supported by the median of three replicates was reported and shown.

### SLAY procedure

SLAY procedures have been detailed previously (*25*, *26*). Briefly, 100 μl of *E. coli* W3110 frozen cells containing the pooled SynCH plasmid library were recovered in 10 ml of LB supplemented with carbenicillin (75 μg/ml) for 2 hours. The culture was then back-diluted to an OD of 0.05, and three triplicate 5-ml cultures were set up in LB supplemented with carbenicillin (75 μg/ml). Triplicate reactions included uninduced (0 μM IPTG), low induction (15 μM IPTG), and high induction (100 μM IPTG). All triplicate cultures were then grown for 3 hours at 37°C. Plasmids from each triplicate culture were isolated via Miniprep, and Illumina sequencing primers (see table S6) were used to produce an amplicon via PCR from plasmids from each culture containing a unique i7 barcode identifier. Amplicons were then gel extracted, cleaned, concentrated, and sent to Genewiz for next-generation sequencing by Illumina HiSeq technology with 30% added Phi-X DNA.

### SLAY sequencing analysis

Methods of SLAY analysis have been previously described elsewhere. Here, raw sequencing reads were trimmed of adapter sequences using Flexbar (*30*) and then assessed for quality via FastQC (*31*). Next, trimmed reads were mapped to a reference derived from all possible SynCH sequences using Kallisto (*32*). Last, log_2_ fold change estimates and other relevant statistics were calculated using DESeq2 (*33*).

### Hemolysis assays

Single-donor human red blood cells (Innovative Research, no. IWB3ALS) were washed in PBS and adjusted to a concentration of 1 × 10^9^ cells/ml. Each peptide was added individually to 200 μl of cells at a concentration of either 300 μg/ml (SySA library) or 128 μg/ml (SySA-5 optimization library) in a 96-well polypropylene plate (Corning, 3879). PBS alone and 1% Triton X-100 were used for background normalization and 100% hemolysis, respectively. Each reaction was set up in triplicate. Plates were incubated for 3 hours at 37°C. Following incubation, samples were centrifuged at 800*g* for 20 min, and 100 μl of supernatant was transferred to a flat-bottom 96-well plate (Genesse, 25-104). Percent hemolysis for each sample was determined by normalizing the absorbance at 540 nm for each sample to the average background and dividing by the average absorbance for 1% Triton X-100 (100% hemolysis). Error bars represent 1 SD of triplicate samples.

### PI uptake

PI uptake was measured for *E. coli* W3110 as previously described. Briefly, single colonies that were from overnight growth on LB were inoculated into MH broth and grown to mid-log phase. Cells were then washed twice with 1× PBS + 50 mM glucose and resuspended to a final optical density of 0.1 in 1× PBS + 50 mM glucose. PI was added to the cells at a concentration of 10 μg/ml (15 μM), and 50 μl of the PI cell mixture was quickly added to a pre-prepared 96-well plate (Nunc black-walled clear bottom plate) that contained 50 μl of different peptide concentrations. The plate had been prepared by serially diluting peptides twofold in 1× PBS + 50 mM glucose; the highest concentration of peptides was equivalent to 128 μg/ml, which, with the addition of cells, became 64 μg/ml. The plate was then allowed to incubate for 25 min in the dark at 37°C. After 25 min had elapsed, the plate was read using a BioTek Synergy LX plate reader with the fluorescence red filter cube every 5 min for 25 min with shaking in between. To account for background fluorescence, each plate contained three triplicate columns with nontreated cells in 1× PBS + 50 mM glucose. Triplicate samples were normalized to nontreated wells and presented as a mean ± 1 SD (*n* = 3).

### GFP release assays

GFP under the control of a strong synthetic Biofab promoter was inserted into the Tn7 site of *E. coli* W3110 as previously described (*34*). Cultures were grown for 16 hours at 37°C, 220 RPMI in LB and diluted in PBS + 50 mM glucose to an OD_600_ of 0.8. Cultures were exposed to SySA peptides (32 μg/ml), positive control compounds (Protegrin-1 at 4 μg/ml and benzalkonium chloride at 16 μg/ml), or negative control compounds (kanamycin at 64 μg/ml and ciprofloxacin at 0.5 μg/ml) for 30 min at 37°C, static. Bacteria were pelleted for 3 min at 13,000*g*, and 100 μl of supernatant was loaded into a black-walled 96-well plate in technical triplicate. Fluorescence intensity was measured at 485-nm excitation and 528-nm emission on a BioTek Synergy H1 with a consistent gain of 100. Fluorescence intensity was calculated by subtracting the mean value from untreated wells from the mean value of treated wells. Three biological replicates are plotted.

### Machine learning and regression modeling

Machine learning models were trained to predict MIC and hemolysis from peptide sequences. For the MIC model, values were first log_2_-transformed. Peptide sequences were embedded as numerical vectors using the Bepler deep protein language model (*35*) from the bio-embeddings Python library. The peptide sequences were split into a training set that represented a random sample 80% the size of the original dataset, and the remaining 20% was set aside as the test dataset. An automated machine learning (autoML) approach (*36*) was used to find the best machine learning regression model that minimized the root mean square error (RMSE). The mljar library was used to carry out the autoML model selection with varying combinations of machine learning architecture, hyperparameters, and feature selection. The combination of model architecture, hyperparameters, and feature selection that minimized RMSE was chosen as the final model.

## Supplementary Materials

### This PDF file includes:

Supplementary Text Figs. S1 to S5 Tables S1 to S7 References

## Notes

View/request a protocol for this paper from *Bio-protocol*.
