# Supplementary material for: Designing and identifying β-hairpin peptide macrocycles with antibiotic potential
Source: Sci Adv. 2023 Jan 11;9(2):eade0008. doi: 10.1126/sciadv.ade0008 (PMC9833666; doi:10.1126/sciadv.ade0008)
Supplement: Supplementary file 1 — Supplementary Text Figs. S1 to S5 Tables S1 to S7 References [file sciadv.ade0008_sm.pdf]

Science Advances
AAAS

Supplementary Materials for

Designing and identifying β-hairpin peptide macrocycles with antibiotic potential

Justin R. Randall *et al.*

Corresponding author: Bryan W. Davies, bwdavies@austin.utexas.edu

Sci. Adv. **9**, eade0008 (2023)
DOI: 10.1126/sciadv.ade0008

The PDF file includes:

Supplementary Text
Figs. S1 to S5
Tables S1 to S7
Legends for data S1 and S2
References

Other Supplementary Material for this manuscript includes the following:

Data S1 and S2

Supplementary Text

SynCH peptide synthesis and SLAY activity

For consistency, all SynCH peptides were resuspended at 10 mg/ml in water. Peptides were then visually inspected for any lack of solubility (Data File S1). SLAY identifies plasmids expressing peptide fusions which slow bacterial growth. Many peptides demonstrating solubility issues were also not found to be active *in vitro*. It is possible these peptides or others slowed bacterial growth in SLAY by aggregating intracellularly before being displayed on the cell surface. To investigate whether soluble SySA peptides may be prone to aggregative qualities, SySA-49 was examined via CD at concentrations ranging from 450 μg/ml to 50 μg/ml (**fig. S4C**). Its molar ellipticity remained relatively constant regardless of increased concentration, suggesting SySA-49 and other soluble peptides are likely not prone to strong aggregation in solution.

SynCH peptide antibiotics can be optimized for therapeutic potential

We questioned if the naïve peptide sequences discovered through our screen could be optimized to improve their activity. Results from our biochemical characterization suggest high charge is important. Previous data from optimization of another SLAY identified β-AMP found that shorter peptide length and additional disulfide bonds increased its potency (*24*). For these reasons we generated a 27-peptide optimization library around our most potent peptide (SySA-5) by shortening its length, increasing its charge, and adding the potential for a second intramolecular disulfide bond while also maintaining alternating residue side chain properties in the antiparallel β-sheets (**table S3**).

We had this library commercially synthesized and tested its ability to inhibit the growth of *Acinetobacter baumannii* AB5075, a clinically isolated Carbapenem Resistant Acinetobacter (CRA) pathogen. MICs were performed in MH as well as 100% FBS, which is likely more representative of the *in vivo* environment. We found that 23 or our 27 variants increased as much as 8-fold in potency in MH. Additionally, eleven gained the ability to inhibit *A. baumanii* AB5075 growth in FBS (**table S3**). This was surprising as degradation in serum is a common limitation of peptide use. Curious whether increased potency correlated with increased toxicity we also measured each variants hemolysis at 128 μg/ml and reported it as a fold change relative to SySA-5 (**table S3**). There was not an equivalent increase in hemolysis for any of the variants. Most had less than a two-fold increase relative to SySA-5. The most potent of the variants (SySA 5.17) had the same MIC against *A. baumanii* AB5075 in FBS as Protegrin-1 (32 μg/ml), but was greater than ten-fold less hemolytic at 128 μg/ml. This data suggests the SynCH peptides can be easily optimized to have greater therapeutic potential than naturally occurring β-AMPs.

Machine learning algorithm and modelling

For this model, MIC values obtained for the top 81 SySA peptides were $\log_2$ transformed and amino acid sequences were embedded as numerical vectors using the Bepler deep protein language model (*35*) from the bio-embeddings python library (*38*). We then used AutoML (*36*) to fit an array of different predictors to 80% of the SySA biochemical data (the training data) and validated performance on the remaining 20% (validation data). Each fitted predictor takes a numerical embedding of a peptide as input and produces a score as output, where a lower score indicates a higher likelihood of antibacterial activity. The AutoML algorithm considered predictors such as linear, random forest, neural networks, or gradient boosting.

The best performing predictor found by AutoML was of type LightGMB (*37*), with 16 selected features. Because the features are embedding scores, we could not interpret them directly. To nonetheless gain some insight into these features, we correlated them with basic properties of peptides such as the proportion of different amino acids, the peptide length, charge, hydrophobicity, and so on. We found that three features perfectly represented the fractions of alanine, proline, and glutamine in a peptide. These and other features did not have a straightforward interpretation or obvious correlations with our library design. Correlations with peptide length, weight, or the amount of cysteine were also common; however, these correlations had relatively low R^2 values of between 10% and 38%. These observations highlight that the protein embedding scores capture non-trivial aspects of peptide biochemistry that don't necessarily correspond to simple and straightforward biochemical quantities (**Fig. 4A**).

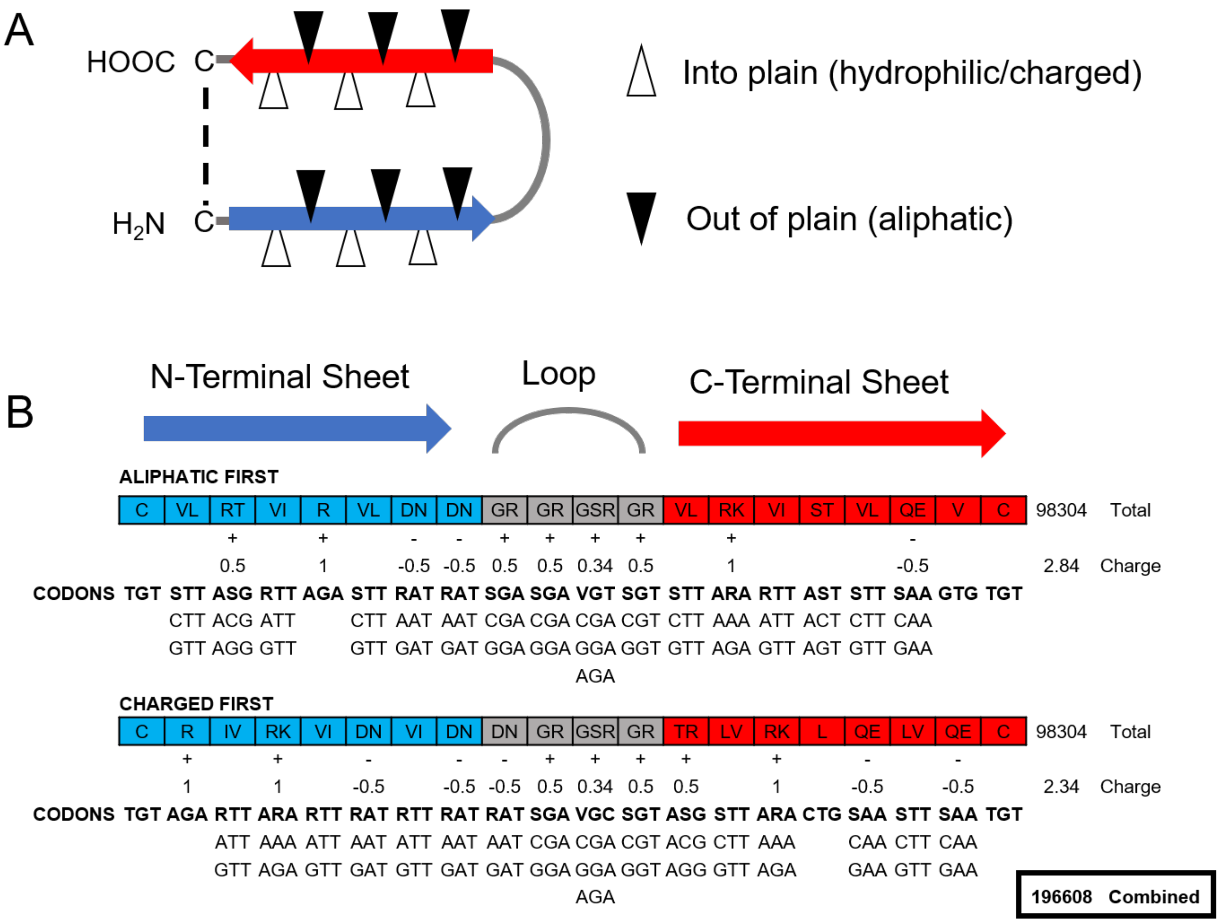

Fig. S1. SynCH peptide library design, Related to Figure 1.
(**A**) Diagram of our basic β-hairpin peptide design. (**B**) Aliphatic first and charged first SynCH libraries showing the separate regions and each potential amino acid at every position. The average charge contribution at each position is shown and the codon variation used to potentiate the different amino acids at each position are below.

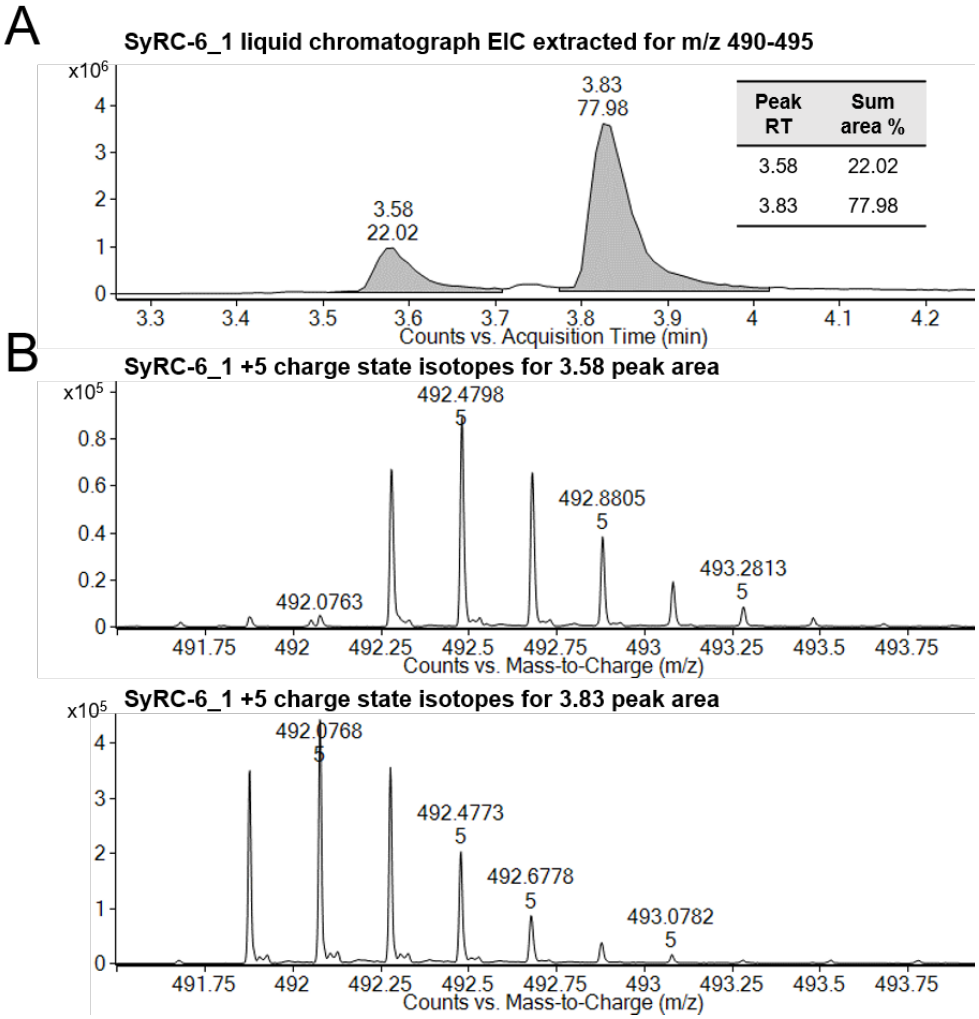

Fig. S2. Calculating peptide percent cyclization, Related to Figure 3E and table S1. (**A**) Liquid chromatography elution peaks from SyRC-6 in PBS. Peak acquisition time and percentage of total elution peaks are in the inset table. (**B**) +5 charge state peaks with the measured monoisotopic mass (M_{mi}) of each peak. Data here represents the measurement of a single sample. the ~2 Dalton drop in monoisotopic mass between the two peaks corresponds to the formation of a disulfide bond.

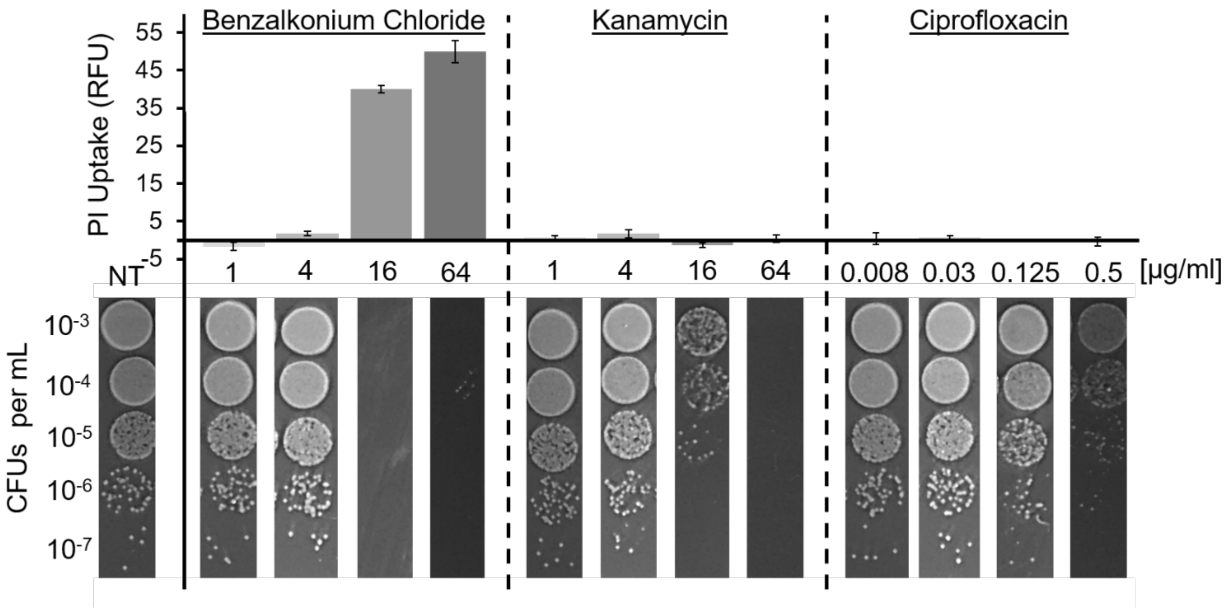

Fig. S3. Propidium iodide uptake controls, Related to Figure 3B. PI uptake and CFUs are shown for treatment of *E. coli* W3110 cells at an OD_{600} = 0.05 with each antimicrobial at the indicated concentrations for 25 minutes. Error bars are one standard deviation of replicates (n=3).

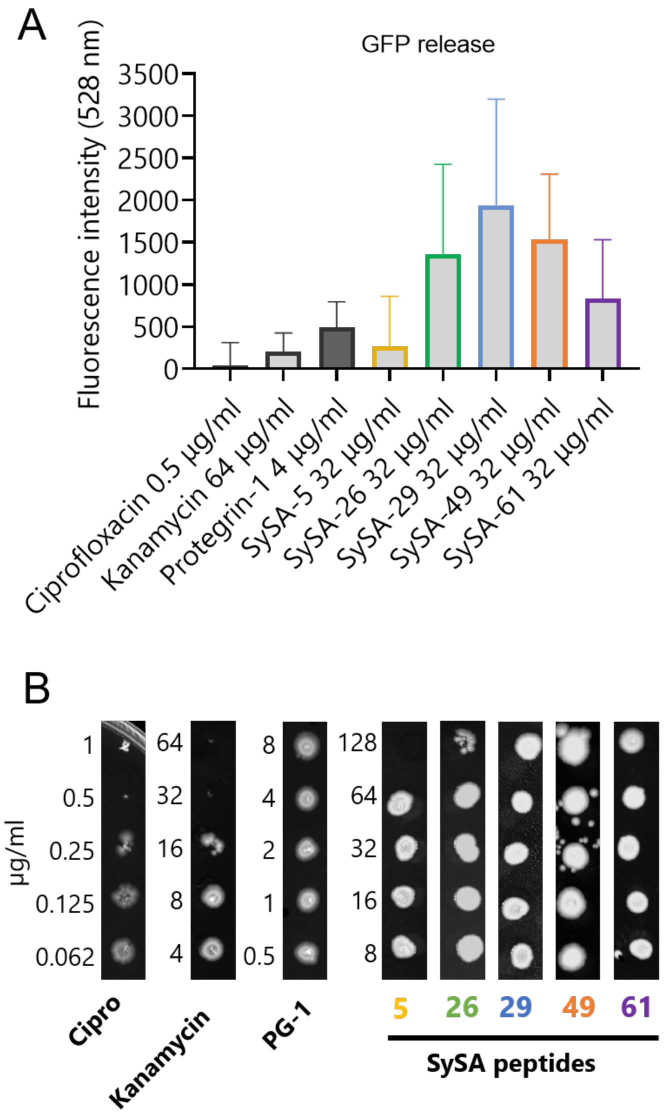

Fig. S4. GFP release from cells treated with SySA peptides, Related to Figure 3B.
(**A**) Fluorescence intensity of GFP released from *E. coli* cells after treatment with antimicrobial peptides for 30 minutes. (**B**) Determination of peptide minimum bactericidal concentrations (MBCs) after overnight treatment at cell densities equivalent to those used in GFP release assays. Error bars represent standard deviation or replicates (n=3).

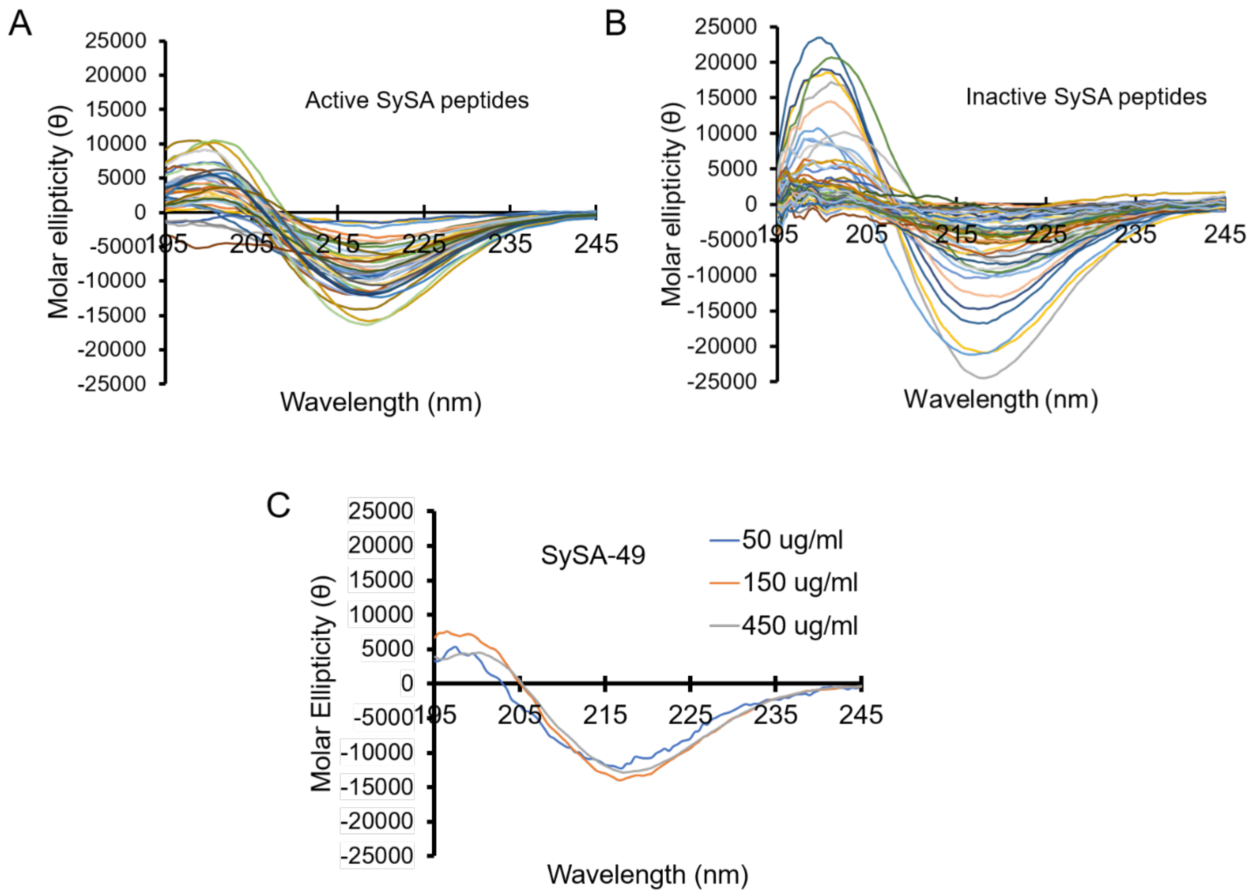

Fig. S5. SynCH SLAY active peptide structures, Related to Figure 3D and table S2. Circular dichroism spectra of the inactive (**A**) and active (**B**) top 88 SySA peptides. C) SySA-49 molar ellipticity spectra at the listed concentrations. Spectra with a single molar ellipticity minimum between 215-220 are considered to contain a β-hairpin secondary structure. Each spectrum is the average of three measurements of the same sample with background removed.

Table S1. Properties of randomly selected peptides from the SynCH library, Related to Figure 1.

Name	Sequence	Charge	GRAVY	β-hairpin CD	%S-S	MIC (µg/ml)
SyRC-1	CRVRINVDDRRGRLKLQVEC	2.88	-0.70	Yes	90.8 ± 2.7	>256
SyRC-2	CRVKINIDNGSRRVRLEVEC	1.88	-0.49	Yes	96.7 ± 0.7	>256
SyRC-3	CRIRVNVNDGSGRVRLQVQC	2.87	-0.33	Yes	21.9 ± 7.8	>256
SyRC-4	CRIRINVNDRGGTVRLEVEC	0.88	-0.31	Yes	100 ± 0.0	>256
SyRC-5	CRVRIDINNGRRRLKLQLQC	4.87	-0.70	Yes	89.7 ± 1.4	>256
SyRC-6	CRIKVNINNRRRTVKLELEC	3.88	-0.67	Yes	80.0 ± 1.7	>256
SyRC-7	CRIKVDIDDRRGRVRLQLEC	1.88	-0.68	Yes	68.7 ± 3.6	>256
SyRC-8	CRIKVNIDDRSRRLRLELEC	1.88	-0.72	Yes	69.2 ± 5.9	>256
SyRC-9	CRVRINVNDGSGTVRLQVQC*	-0.12	-0.31	-	-	-
SyRC-10	CRVRINVNDGSGTVRLQVQC	1.87	-0.15	No	95.7 ± 2.2	>256
SyRC-11	CRVRINVDNRGGTLKLQVQC	2.87	-0.32	Yes	89.5 ± 1.0	>256
SyRC-12	CRIRINVDNGGGTVRLQVEC	0.88	-0.12	Yes	nd	>256
SyRC-13	CRIRIDIDDRSRTLKLQLQC	1.88	-0.52	Yes	8.2 ± 3.6	>256
SyRC-14	CRVRINVNNRSGRLKLQVEC	3.88	-0.52	Yes	100 ± 0.0	>256
SyRC-15	CRVKVNVDDRGRRLKLELQC	2.88	-0.70	Yes	79.1 ± 3.0	>256
SyRA-1	CLRVRLNNGGRRVKVSVQVC*	1.88	-0.04	-	-	-
SyRA-2	CLRIRVNNRRGGVRITVQVC	4.87	0.00	Yes	100 ± 0.0	>256
SyRA-3	CLRVRLNNGGRRVKVSVQVC	4.87	-0.02	No	77.9 ± 2.0	256
SyRA-4	CLTIRVDNRRSRVKITLEVC	2.88	-0.02	Yes	85.2 ± 2.2	>256
SyRA-5	CLTIRLDNGGRGVRISLQVC	1.87	0.34	No	nd	>256
SyRA-6	CVRVRLNDGGSGVRISVQVC	1.87	0.36	Yes	78.4 ± 6.3	>256
SyRA-7	CVTIRLNDRRRGVKISVEVC	2.88	0.01	No	66.3 ± 8.2	>256
SyRA-8	CLRIRVNNGGSRVRISLEVC*	0.88	0.41	-	-	-
SyRA-9	CVTIRLDDRRRGLRVTVQVC	2.87	-0.04	Yes	94.3 ± 1.2	>256
SyRA-10	CLRIRVNNGGSRVRISLEVC	2.88	0.16	Yes	66.2 ± 11.9	>256
SyRA-11	CVTIRVDNRGSRVRVTLEVC	1.88	0.15	Yes	0.0 ± 0.0	>256
SyRA-12	CVRVRVDDRRGGLKISLQVC	2.87	0.00	Yes	100 ± 0.0	>256
SyRA-13	CVTVRLDNGRSGVRISVQVC*	1.87	0.34	-	-	-
SyRA-14	CVTVRVNNRGGRLKITLQVC	3.87	0.18	Yes	100 ± 0.0	>256
SyRA-15	CVTVRLNDRRSRLRITLEVC	2.88	-0.08	Yes	89.3 ± 2.3	>256

* was not synthesized, GRAVY: grand average of hydropathicity, β-hairpin CD was determined by having an ellipticity minimum between 215 and 220 nm, %S-S: percentage of peptide with a disulfide bond represented by the mean ± one standard deviation of three technical replicates, MIC: minimum inhibitory concentration, nd: not determined

Table S2. Properties of SynCH peptides verified as antibacterial, Related to Figure 3.

Name	Sequence	W3110 MIC (μg/ml)		Hemolysis (%)	β-hairpin CD
		MH	RPMI		
SySA-1	CRIKVDVNNRRRRVRLQVQC	128	64	0.57 ± 0.23	Yes
SySA-4	CRIKVNVNNRGRRLRLEVQC	256	128	0.81 ± 0.56	Yes
**SySA-5**	CLRVRLNNRRGRVKVSLQVC	16	4	3.73 ± 1.28	Yes
SySA-7	CRVRININNGRRRVKLQLQC	256	32	0.50 ± 0.26	Yes
SySA-8	CRIKINVNDGSRRLKLQVQC	256	256	0.23 ± 0.19	Yes
**SySA-9**	CVRVRLNNRRRGLKISLQVC	128	8	1.13 ± 0.80	Yes
**SySA-11**	CVTVRVNNRRRRVRISVQVC	64	16	0.66 ± 0.12	Yes
SySA-16	CVRVRLNNRRRRLKVSLEVC	32	32	3.24 ± 1.16	Yes
**SySA-17**	CLRVRLNNRRRGLKVSVQVC	64	32	0.84 ± 0.18	Yes
**SySA-25**	CLRVRLNNRRRGVKVSVQVC	64	8	0.77 ± 0.53	Yes
**SySA-26**	CVRVRLNNGRRRVKVSLQVC	16	16	1.05 ± 0.31	Yes
SySA-27	CVRVRLNNRGGGLRITLQVC	256	64	3.40 ± 0.87	Yes
**SySA-29**	CVRVRLNNRRRGLRVSLQVC	16	8	1.22 ± 0.41	Yes
**SySA-30**	CLRIRLNNRRRGLKVSLQVC	64	8	0.61 ± 0.23	Yes
SySA-34	CRIKVNINNGRRRVKLQLQC	128	32	0.23 ± 0.07	Yes
SySA-37	CVRIRLNNRRRRVKVSLEVC	32	8	2.84 ± 0.49	Yes
SySA-38	CVRIRVNNNRRSGVKVSLQVC	256	8	0.30 ± 0.41	Yes
**SySA-40**	CVRIRLNNRRRGLKVSLQVC	32	8	0.61 ± 0.36	Yes
**SySA-45**	CVRVRLNNRRRGLKVTLQVC	256	32	7.74 ± 1.14	No
**SySA-49**	CVRVRLNNRRRGVKISLQVC	32	8	0.29 ± 0.49	Yes
SySA-53	CVRIRLNNGGRRVKVTLQVC	32	32	0.71 ± 0.55	Yes
**SySA-61**	CLRVRLNNRRRGLKISLQVC	32	8	1.55 ± 0.74	Yes
SySA-62	CVRIRVNNRRRRVKVSVQVC	32	16	1.36 ± 0.74	Yes
SySA-63	CVTIRVNNNRRSRVRISLQVC	256	64	0.79 ± 0.73	Yes
SySA-65	CVRVRLNNRRRRVKISVEVC	64	16	5.19 ± 1.35	Yes
SySA-66	CVRIRLNNRRRGVKVSVQVC	128	32	0.52 ± 0.53	Yes
**SySA-71**	CLRVRLNNGRRRLKVSLQVC	16	32	1.74 ± 0.57	Yes
**SySA-72**	CLRIRLNNRRRGLRISLQVC	32	8	3.17 ± 0.40	Yes
**SySA-73**	CLRVRLNNRRRGLRISLQVC	32	8	3.02 ± 0.82	Yes
SySA-78	CVRVRLNNRRRRLKISLEVC	32	16	6.59 ± 2.26	Yes
**SySA-79**	CVRVRVNNGRRRLKVSLQVC	64	32	1.19 ± 0.79	Yes
SySA-80	CVRIRLNNRRRRVKVTLQVC	16	16	2.93 ± 1.04	Yes
SySA-81	CLTVRLNNRRRGVKVSVQVC	64	32	0.76 ± 0.24	Yes
**SySA-82**	CVRIRLNNRRRGLKISLQVC	64	8	1.11 ± 0.61	Yes
SySA-83	CVRVRVNNRGGRLKISVQVC	128	16	0.59 ± 0.32	Yes
**SySA-88**	CVRVRLNNRRRGLKITLQVC	32	8	3.43 ± 0.72	Yes
Thanatin	GSKKPVPIIYCNRRTGKCQRM	2	4	0.30 ± 0.87	-
Protegrin-1	RGGRLCYCRRRFCVCVGR	0.5	0.5	41.84 ± 5.03	-

MIC: minimum inhibitory concentration, MH: Mueller-Hinton, RPMI: RPMI 11640; β-hairpin spectrum was determined by a circular dichroism ellipticity minimum between 215 and 220 nm. **Bold:** part of the same charge-GRAVY score grouping.

Table S3. SySA-5 *A. baumannii* AB5075 optimization library, Related to Figure 3.

Name	Sequence	AB5075 MIC (µg/ml) MH	AB5075 MIC (µg/ml) FBS	Length (AAs)	Potential Cysteine pairs	# Cationic AAs	Fold Change Hemolysis
SySA-5	CLRVRLNNRRGRVKVSLQVC	32	>128	20	1	6	1.0
SySA-5.1	CLRVRLRNRRGRVKVSLRVC	8	128	20	1	8	1.8
SySA-5.2	CLRVRLRNRRRRVKVRLRVC	4	>128	20	1	10	2.6
SySA-5.3	CLRVRLCNRRRCVKVRLRVC	8	128	20	2	8	1.8
SySA-5.4	CLRVRLCRRRRCVKVRLRVC	8	64	20	2	10	2.9
SySA-5.5	C-RVRLNNRRGRVKVSLQ-C	8	>128	18	1	6	0.9
SySA-5.6	C-RVRLRNRRGRVKVSLR-C	4	128	18	1	8	0.7
SySA-5.7	C-RVRLRNRRRRVKVRLR-C	8	>128	18	1	10	0.9
SySA-5.8	C-RVRLCNRRRCVKVSLR-C	8	>128	18	2	7	0.8
SySA-5.9	C-RVRLCRRRRCVKVRLR-C	8	>128	18	2	9	0.6
SySA-5.10	CLRVRLN-RRGR-KVSLQVC	4	128	18	1	6	1.4
SySA-5.11	CLRVRLR-RRGR-KVRLQVC	4	64	18	1	8	3.0
SySA-5.12	CLRVRLR-RRRR-KVRLRVC	4	>128	18	1	10	3.7
SySA-5.13	CLRVRLC-RRGR-CVSLRVC	16	>128	18	2	6	3.2
SySA-5.14	CLRVRLC-RRRR-CVRLRVC	4	64	18	2	8	5.6
SySA-5.15	C-RVRLNCRRGRCKVSLQ-C	16	>128	18	2	6	1.3
SySA-5.16	C-RVRLRCRRGRCKVSLR-C	32	>128	18	2	8	1.3
SySA-5.17	C-RVRLRCRRRRCKVRLR-C	4	32	18	2	10	2.4
SySA-5.18	CLRVRLN-RR-R-KVSLQVC	8	128	17	1	6	1.0
SySA-5.19	CLRVRLR-RR-R-KVRLQVC	4	>128	17	1	8	2.5
SySA-5.20	CLRVRLR-RR-R-KVRLRVC	4	128	17	1	9	3.3
SySA-5.21	CLRVRLC-RR-R-CVSLRVC	16	>128	17	2	6	2.4
SySA-5.22	CLRVRLC-RR-R-CVRLRVC	32	64	17	2	7	2.1
SySA-5.23	C-RVRLN-RRGR-KVSLQ-C	8	>128	16	1	6	1.0
SySA-5.24	C-RVRLR-RRGR-KVSLR-C	16	>128	16	1	8	1.1
SySA-5.25	C-RVRLR-RRRR-KVRLR-C	32	>128	16	1	10	1.5
SySA-5.26	C-RVRLC-RRRR-CVRLR-C	8	>128	16	2	8	0.8
SySA-5.27	C-RVRCR-RRRR-KCRLR-C	128	>128	16	2	10	0.6
Protegrin-1	RGGRLCYCRRRFCVCVGR	1	32	18	2	6	32.7

MIC: minimum inhibitory concentration, MH: Mueller-Hinton, FBS: fetal bovine serum, AA: amino acid; **bold:** residue change; green: fold-improvement, red: fold-deterioration

Table S4. SySA-5.17 activity spectrum.

Bacterial Strain	SySA 5.17 MIC in MH (µg/ml)
A. baumannii AB5075*	4
V. cholerae C6706	16
P. aeruginosa ATCC 27853	16
K. pneumonia ATCC 1705*	64
S. aureus USA100*	8
E. coli W3110	4
E. coli WD101^	4
A. baumannii 17978	8
A. baumannii 17978 R2^	8

* Denotes multidrug resistance; ^ Denotes colistin resistance
MH = Standard Mueller-Hinton media

Table S5. Colistin resistant strains.

Bacterial Strain	Colistin MIC in MH (µg/ml)
E. coli W3110	0.125
E. coli WD101^	4
A. baumannii 17978	0.25
A. baumannii 17978 R2^	16

^ Denotes colistin resistance
MH = Standard Mueller-Hinton media

Table S6. Plasmids and Oligonucleotides.

Plasmids	Source
pMMBEH67_lpp_ompA	(*26*)
**Oligonucleotides**	**Sequence**
oJR557 - F SynCH	gtattggtaccagtcaagagcctg
oJR560 - R SynCH Aliphatic First	ctg cag gtc gac tta ACA CAC TTS AAS AST AAY TYT AAS ACS ACB TCS TCS ATY ATY AAS TCT AAY CST AAS ACA ggt tcc tcc gat acc cgc ag
oJR561 - R SynCH Charged First	ctg cag gtc gac tta ACA TTS AAS TTS CAG TYT AAS CST ACS GCB TCS ATY ATY AAY ATY AAY TYT AAY TCT ACA ggt tcc tcc gat acc cgc ag
2x(NR)tether gBlock	ATTGCCGATGGTACACGTCAAGTCAAGAGCCTGCAGCGCCCGCCGCAGAGGCGACTCCTGCTGCTGAAGCTCCAGCTAGCGAAGCGCCTGCAGCAGAAGCTGCCCCAGCGGATGCTGCCGAAGCCCCAGCCGCTGGCATCAGTCAGGAACCTGCTGCACCAGCTGCGGAAGCTACACCAGCAGCGGAGGCACCAGCGAGTGAAGCACCGGCTGCGGAAGCCGCTCCTGCAGATGCCGCTGAGGCTCCAGCTGCGGGTATCGGAGGAACCCGCGGTGGGCGTCTTTGTTA
F amplicon	aatgATACGGCGACCACCGAGATCTACACTCTTTCCCTACACGACGCTCTTCCGATCTCTCCAGCTGCGGGTATCGGAGGA
R index 1	CAAGCAGAAGACGGCATACGAGATCGTGATGTGACTGGAGTTCAGACGTGTGCTCTTCCGATCTgccaagcttgcatgcctgcaggtcgacTTA
R index 2	CAAGCAGAAGACGGCATACGAGATACATCGGTGACTGGAGTTCAGACGTGTGCTCTTCCGATCTgccaagcttgcatgcctgcaggtcgacTTA
R index 3	CAAGCAGAAGACGGCATACGAGATGCCTAAGTGACTGGAGTTCAGACGTGTGCTCTTCCGATCTgccaagcttgcatgcctgcaggtcgacTTA
R index 4	CAAGCAGAAGACGGCATACGAGATTGGTCAGTGACTGGAGTTCAGACGTGTGCTCTTCCGATCTgccaagcttgcatgcctgcaggtcgacTTA
R index 5	CAAGCAGAAGACGGCATACGAGATCACTGTGTGACTGGAGTTCAGACGTGTGCTCTTCCGATCTgccaagcttgcatgcctgcaggtcgacTTA
R index 6	CAAGCAGAAGACGGCATACGAGATATTGGCGTGACTGGAGTTCAGACGTGTGCTCTTCCGATCTgccaagcttgcatgcctgcaggtcgacTTA
R index 7	CAAGCAGAAGACGGCATACGAGATGATCTGGTGACTGGAGTTCAGACGTGTGCTCTTCCGATCTgccaagcttgcatgcctgcaggtcgacTTA
R index 8	CAAGCAGAAGACGGCATACGAGATTCAAGTGTGACTGGAGTTCAGACGTGTGCTCTTCCGATCTgccaagcttgcatgcctgcaggtcgacTTA
R index 9	CAAGCAGAAGACGGCATACGAGATCTGATCGTGACTGGAGTTCAGACGTGTGCTCTTCCGATCTgccaagcttgcatgcctgcaggtcgacTTA
R index 10	CAAGCAGAAGACGGCATACGAGATAAGCTAGTGACTGGAGTTCAGACGTGTGCTCTTCCGATCTgccaagcttgcatgcctgcaggtcgacTTA
R index 11	CAAGCAGAAGACGGCATACGAGATGTAGCCGTGACTGGAGTTCAGACGTGTGCTCTTCCGATCTgccaagcttgcatgcctgcaggtcgacTTA
R index 12	CAAGCAGAAGACGGCATACGAGATTACAAGGTGACTGGAGTTCAGACGTGTGCTCTTCCGATCTgccaagcttgcatgcctgcaggtcgacTTA

All oligonucleotides were ordered from Integrated DNA technologies (IDT). IDT single letter nucleotide base notations are used.

Table S7: Strains used.

Strains	Source
E. coli W3110	Lab Stock
E. coli WD101	Lab Stock
A. baumannii AB5075	(*39*)
A. baumannii 17978	Lab Stock
A. baumannii 17978 R2	Lab Stock
V. cholerae C6706	Lab Stock
P. aeruginosa ATCC 27853	ATCC
K. pneumonia ATCC 1705	ATCC
S. aureus USA100	Lab Stock

Data S1. (separate file)

Excel spreadsheet with chemically synthesized peptide properties and biochemical data.

Data S2. (separate file)

Excel spreadsheet with all SynCH SLAY data analysis and potency score predictions

REFERENCES AND NOTES

1. P. Morales, M. A. Jiménez, Design and structural characterisation of monomeric water-soluble α-helix and β-hairpin peptides: State-of-the-art. *Arch. Biochem. Biophys.* **661**, 149–167 (2019).

2. M. Angeles Jiménez, Design of monomeric water-soluble β-hairpin and β-sheet peptides. *Methods Mol. Biol.* **1216**, 15–52 (2014).

3. F. J. Blanco, G. Rivas, L. Serrano, A short linear peptide that folds into a native stable β-hairpin in aqueous solution. *Nat. Struct. Biol.* **1**, 584–590 (1994).

4. J. S. Richardson, D. C. Richardson, Natural beta-sheet proteins use negative design to avoid edge-to-edge aggregation. *Proc. Natl. Acad. Sci. U.S.A.* **99**, 2754–2759 (2002).

5. J. R. Randall, B. W. Davies, Mining for novel antibiotics. *Curr. Opin. Microbiol.* **63**, 66–69 (2021).

6. A. Luther, C. Bisang, D. Obrecht, Advances in macrocyclic peptide-based antibiotics. *Bioorg. Med. Chem.* **26**, 2850–2858 (2018).

7. I. Martin-Loeches, G. E. Dale, A. Torres, Murepavadin: A new antibiotic class in the pipeline. *Expert Rev. Anti. Infect. Ther.* **16**, 259–268 (2018).

8. P. V. Panteleev, S. V. Balandin, V. T. Ivanov, T. V. Ovchinnikova, A therapeutic potential of animal β-hairpin antimicrobial peptides. *Curr. Med. Chem.* **24**, (2017).

9. K. L. H. Lam, Y. Ishitsuka, Y. Cheng, K. Chien, A. J. Waring, R. I. Lehrer, K. Y. C. Lee, Mechanism of supported membrane disruption by antimicrobial peptide protegrin-1. *J. Phys. Chem. B* **110**, 21282–21286 (2006).

10. S. U. Vetterli, K. Zerbe, M. Müller, M. Urfer, M. Mondal, S.-Y. Wang, K. Moehle, O. Zerbe, A. Vitale, G. Pessi, L. Eberl, B. Wollscheid, J. A. Robinson, Thanatin targets the intermembrane protein complex required for lipopolysaccharide transport in Escherichia coli. *Sci. Adv.* **4**, eaau2634 (2018).

11. P. V. Panteleev, I. A. Bolosov, S. V. Balandin, T. V. Ovchinnikova, Structure and biological functions of β-hairpin antimicrobial peptides. *Acta Naturae* **7**, 37–47 (2015).

12. I. A. Edwards, A. G. Elliott, A. M. Kavanagh, J. Zuegg, M. A. T. Blaskovich, M. A. Cooper, Contribution of amphipathicity and hydrophobicity to the antimicrobial activity and cytotoxicity of β-hairpin peptides. *ACS Infect Dis.* **2**, 442–450 (2016).

13. I. A. Edwards, A. G. Elliott, A. M. Kavanagh, M. A. T. Blaskovich, M. A. Cooper, Structure-activity and -toxicity relationships of the antimicrobial peptide tachyplesin-1. *ACS Infect Dis.* **3**, 917–926 (2017).

14. C. D. DuPai, B. W. Davies, C. O. Wilke, A systematic analysis of the beta hairpin motif in the Protein Data Bank. *Protein Sci.* **30**, 613–623 (2021).

15. K. Fujiwara, H. Toda, M. Ikeguchi, Dependence of α-helical and β-sheet amino acid propensities on the overall protein fold type. *BMC Struct. Biol.* **12**, 18 (2012).

16. J. M. Otaki, M. Tsutsumi, T. Gotoh, H. Yamamoto, Secondary structure characterization based on amino acid composition and availability in proteins. *J. Chem. Inf. Model.* **50**, 690–700 (2010).

17. A. M. C. Marcelino, L. M. Gierasch, Roles of beta-turns in protein folding: From peptide models to protein engineering. *Biopolymers* **89**, 380–391 (2008).

18. S. R. Trevino, S. Schaefer, J. M. Scholtz, C. N. Pace, Increasing protein conformational stability by optimizing beta-turn sequence. *J. Mol. Biol.* **373**, 211–218 (2007).

19. S. N. Malkov, M. V. Živković, M. V. Beljanski, M. B. Hall, S. D. Zarić, A reexamination of the propensities of amino acids towards a particular secondary structure: Classification of amino acids based on their chemical structure. *J. Mol. Model.* **14**, 769–775 (2008).

20. S. Costantini, G. Colonna, A. M. Facchiano, Amino acid propensities for secondary structures are influenced by the protein structural class. *Biochem. Biophys. Res. Commun.* **342**, 441–451 (2006).

21. C. M. Santiveri, M. A. Jiménez, Tryptophan residues: Scarce in proteins but strong stabilizers of β-hairpin peptides. *Biopolymers* **94**, 779–790 (2010).

22. N. Greenfield, G. D. Fasman, Computed circular dichroism spectra for the evaluation of protein conformation. *Biochemistry* **8**, 4108–4116 (1969).

23. C. Avitabile, L. D. D'Andrea, A. Romanelli, Circular dichroism studies on the interactions of antimicrobial peptides with bacterial cells. *Sci. Rep.* **4**, 4293 (2014).

24. J. R. Randall, G. Davidson, R. M. Fleeman, S. A. Acosta, I. M. Riddington, T. J. Cole, C. D. DuPai, B. W. Davies, Synthetic antibacterial discovery of symbah-1, a macrocyclic β-hairpin peptide antibiotic. *iScience* **25**, 103611 (2022).

25. A. T. Tucker, S. P. Leonard, C. D. DuBois, G. A. Knauf, A. L. Cunningham, C. O. Wilke, M. S. Trent, B. W. Davies, Discovery of next-generation antimicrobials through bacterial self-screening of surface-displayed peptide libraries. *Cell* **172**, 618–628.e13 (2018).

26. J. R. Randall, C. D. DuPai, B. W. Davies, Discovery of antimicrobial peptide macrocycles through bacterial display. *Methods Mol. Biol.* **2371**, 287–298 (2022).

27. C. Jeworrek, F. Evers, J. Howe, K. Brandenburg, M. Tolan, R. Winter, Effects of specific versus nonspecific ionic interactions on the structure and lateral organization of lipopolysaccharides. *Biophys. J.* **100**, 2169–2177 (2011).

28. N. Soundrarajan, S. Park, Q. le Van Chanh, H. sun Cho, G. Raghunathan, B. Ahn, H. Song, J. H. Kim, C. Park, Protegrin-1 cytotoxicity towards mammalian cells positively correlates with the magnitude of conformational changes of the unfolded form upon cell interaction. *Sci. Rep.* **9**, 11569 (2019).

29. J. M. Anderson, B. Jurban, K. N. L. Huggins, A. A. Shcherbakov, I. Shu, B. Kier, N. H. Andersen, Nascent hairpins in proteins: Identifying turn loci and quantitating turn contributions to hairpin stability. *Biochemistry* **55**, 5537–5553 (2016).

30. J. T. Roehr, C. Dieterich, K. Reinert, Flexbar 3.0–SIMD and multicore parallelization. *Bioinformatics* **33**, 2941–2942 (2017).

31. Babraham Bioinformatics, FastQC: A quality control tool for high throughput sequence data; www.bioinformatics.babraham.ac.uk/projects/fastqc.

32. N. L. Bray, H. Pimentel, P. Melsted, L. Pachter, Near-optimal probabilistic RNA-seq quantification. *Nat. Biotechnol.* **34**, 525–527 (2016).

33. M. I. Love, W. Huber, S. Anders, Moderated estimation of fold change and dispersion for RNA-seq data with DESeq2. *Genome Biol.* **15**, 1–21 (2014).

34. D. A. I. Mavridou, D. Gonzalez, W. Kim, S. A. West, K. R. Foster, Bacteria use collective behavior to generate diverse combat strategies. *Curr. Biol.* **28**, 345–355.e4 (2018).

35. A. Tristan Bepler, B. Berger, T. Bepler, Learning the protein language: Evolution, structure, and function. *Cell Syst.* **12**, 654–669.e3 (2021).

36. G. Luo, B. L. Stone, M. D. Johnson, P. Tarczy-Hornoch, A. B. Wilcox, S. D. Mooney, X. Sheng, P. J. Haug, F. L. Nkoy, Automating construction of machine learning models with clinical big data: Proposal rationale and methods. *JMIR Res. Protoc.* **6**, e175 (2017).

37. G. Ke, Q. Meng, T. Finley, T. Wang, W. Chen, W. Ma, Q. Ye, T.-Y. Liu, LightGBM: A highly efficient gradient boosting decision tree, *Proceedings of the 31st International Conference on Neural Information Processing Systems*, December 2017, pp. 3149–3157.

38. C. Dallago, K. Schütze, M. Heinzinger, T. Olenyi, M. Littmann, A. X. Lu, K. K. Yang, S. Min, S. Yoon, J. T. Morton, B. Rost, Learned embeddings from deep learning to visualize and predict protein sets. *Curr. Protoc.* **1**, e113 (2021).
